# Identification of protein complexes and functional modules in *E. coli* PPI networks

**DOI:** 10.1186/s12866-020-01904-6

**Published:** 2020-08-06

**Authors:** Ping Kong, Gang Huang, Wei Liu

**Affiliations:** 1 Shanghai Key Laboratory of Molecular Imaging, Shanghai University of Medicine and Health Sciences, Shanghai, 201318 China; 2 Research Center for Intelligence Information Technology, Nantong University, Nantong, 226019 Jiangsu China; 3 School of Mathematics and Statistics Science, Lu Dong University, Yantai, 264025 Shandong China

**Keywords:** Protein complexes, Functional modules, Link clustering algorithm, *E. coli* PPI networks

## Abstract

**Background:**

*Escherichia coli* always plays an important role in microbial research, and it has been a benchmark model for the study of molecular mechanisms of microorganisms. Molecular complexes, operons, and functional modules are valuable molecular functional domains of *E. coli*. The identification of protein complexes and functional modules of *E. coli* is essential to reveal the principles of cell organization, process, and function. At present, many studies focus on the detection of *E. coli* protein complexes based on experimental methods. However, based on the large-scale proteomics data set of *E. coli,* the simultaneous prediction of protein complexes and functional modules, especially the comparative analysis of them is relatively less.

**Results:**

In this study, the Edge Label Propagate Algorithm (ELPA) of the complex biological network was used to predict the protein complexes and functional modules of two high-quality PPI networks of *E. coli*, respectively. According to the gold standard protein complexes and function annotations provided by EcoCyc dataset, most protein modules predicted in the two datasets matched highly with real protein complexes, cellular processes, and biological functions. Some novel and significant protein complexes and functional modules were revealed based on ELPA. Moreover, through a comparative analysis of predicted complexes with corresponding functional modules, we found the protein complexes were significantly overlapped with corresponding functional modules, and almost all predicted protein complexes were completely covered by one or more functional modules. Finally, on the same PPI network of *E. coli*, ELPA was compared with a well-known protein module detection method (MCL) and we found that the performance of ELPA and MCL is comparable in predicting protein complexes.

**Conclusions:**

In this paper, a link clustering method was used to predict protein complexes and functional modules in PPI networks of *E. coli*, and the correlation between them was compared, which could help us to understand the molecular functional units of *E. coli* better.

## Background

*Escherichia coli* (*E. coli*) is the primary model organism of microorganisms, and perhaps it is the most intensively studied species of bacteria [1–4]. Even so, only two-thirds of the protein-coding gene products of *E. coli* K-12 currently have experimental evidence for their biological roles, and others remain unannotated (orphans) [5]. Experiments and data analysis (algorithmic model) are two effective methods to identify protein complexes and functional modules of *E. coli*. However, it is well-known that experimental analysis has always been dominant because of the lack of large-scale experimental data and the incompleteness of the *E. coli* dataset [5–9]. Experimental methods have the advantages of direct verification, but they also have the limitations of high false positive rates and false negative rates. In recent years, with the development of genomic technology, some high-throughput, high-quality, binary protein interaction (PPIs) maps of *E. coli* have been released, so the protein complexes and functional modules of *E. coli* and their relationships can be predicted from a global perspective [8–19]. Although the analysis methods of these data were not perfect, many studies showed that the prediction results of these methods are useful supplements to the experimental methods.

A protein complex is formed by the interaction of more than two functional related peptide chains through disulfide bonds or other proteins, so it performs some given biological functions. A functional module is the basic functional unit of proteins, which implies complex relationships involving multiple biological interaction types [8]. Revealing protein complexes and functional modules in the *E. coli* PPI network is an important research topic to understand the essential biological functions of proteins. Although some studies used complex network models to make predictions, due to the lack of large-scale PPI datasets and the existence of a large number of orphan proteins, the prediction results were difficult to achieve the expected ones. However, with the release of some high-throughput *E. coli* PPI datasets in recent years, it has become possible to predict protein complexes and functional modules of *E. coli* based on complex network models. With the development of high-throughput sequencing technologies, such as two-hybrid systems and mass spectrometry technology for pairwise protein interactions, large-scale PPI networks of *E. coli* can be constructed at genome level [20]. Some studies explored the prediction of protein complexes based on *E. coli* PPI networks [5, 10, 11], and others focused on the functional relationship between transcription regulation [12–16] and metabolic pathway [17–19] of *E. coli*. Even so, studies of predicting protein complexes and functional modules at the same time, especially the comparative analysis of them is relatively less. In this paper, based on the *E. coli* datasets of Hu et al. [5] and Cong et al. [8], two complex-related PPI networks were constructed, named netH and netC, respectively. We focused on the recognition and analysis of protein complexes and functional modules in the two large-scale PPI networks, and discussed the differences and connections between them.

Node clustering and link clustering are two different methods to reveal the network structure from different perspectives. Because the link itself contains node attributes, link clustering has a natural advantage over the node clustering algorithm in the identification of network modules. In this study, *E. coli* protein complexes and functional modules were predicted by a link clustering method (ELPA [21]) in two high-quality PPI networks. Many studies showed that Markov Clustering algorithm (MCL) was an excellent protein module identification algorithm and the most popular method for detecting protein complexes [22–25], so we compared the results of ELPA with MCL on the same PPI network of *E. coli.* According to the gold-standard protein complexes and function annotations provided by EcoCyc dataset, the results showed that most protein modules predicted by ELPA matched well with real protein complexes, cellular processes, or biological functions, and the performance was comparable with MCL. For example, in the PPI network of *E. coli* provided by Hu et al., 75.8% of predicted protein modules matched with one or more real protein complexes, 88.1% of real protein complexes matched with one or more protein modules, and 88.3% of predicted protein modules matched with at least one functional unit of *E. coli*. Furthermore, some novel protein complexes and functional modules were uncovered in both networks, and we compared the protein complex with the corresponding functional module predicted from the same protein module. The results showed that the protein complex significantly overlapped with the corresponding functional module, and many functional modules contained more than one protein complex. Therefore, we concluded that ELPA is an effective method to predict protein complexes and functional modules in PPI networks of *E. coli*.

## Results

### Identification of protein modules

ELPA predicted 120 and 171 protein modules in netH and netC (Table S1), respectively. The size of predicted modules ranged from two to hundreds of proteins. Also, we found that many protein modules detected by ELPA overlapped each other. This phenomenon was very consistent with real protein complexes and functional modules, which meant that some proteins involved multiple complexes or functional modules. It is also an important research topic to study the overlapping proteins in different complexes or functional modules.

### Prediction of protein complexes

Those protein modules of netH and netC identified by ELPA were matched with 295 real benchmark protein complexes of *E. coli* in the EcoCyc dataset, respectively. In netH, 222 benchmark complexes (75.3%) matched 91 predicted protein modules (75.8%), and in netC, 200 benchmark complexes (67.8%) matched 144 predicted protein modules (70.9%). Since most benchmark complexes consist of no more than ten proteins, larger protein modules may contain multiple complexes, which is consistent with the composition of real complexes.

**Fig. 1:**
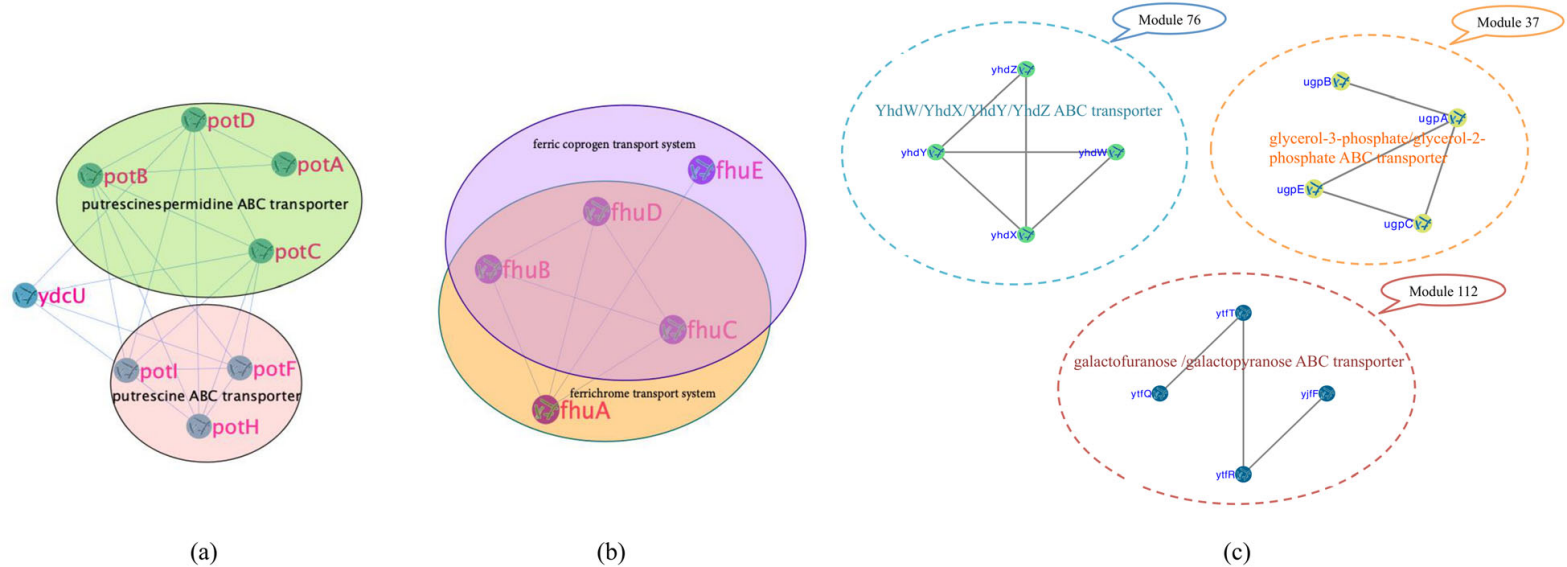
Predicted protein complexes in netH. **a** The proteins of module 50 in netH matched with real complexes. **b** The proteins of module 54 in netH matched with real complexes. **c** Three predicted modules perfectly matched with the corresponding protein complexes in netC

When the Matching Score threshold was set to 0.2 [24, 26], 84 and 136 protein complexes were predicted in netH and netC, respectively. By comparing with the benchmark complexes, we found that most of the protein complexes predicted by ELPA matched well with the corresponding real complexes in both networks. For example, in netH, module 50 consisted of eight proteins, of which *potF, potH*, and *potI* were the three proteins in the putrescine ABC transporter complex; *potA, potB, potC,* and *potD* covered all four proteins of the putrescine/spermidine ABC transporter complex (as shown in Fig. 1a). Module 54 protein module consisted of five proteins, which were completely covered by two complexes: the ferrichrome transport system and the ferric coprogen transport system. The ferrichrome transport system protein complex consisted of four proteins: *fhuA, fhuB, fhuC*, and *fhuD*, while the ferric coprogen transport system protein complex was composed of *fhuB, fhuC, fhuD*, and *fhuE*. As shown in Fig. 1b, it could be found that *fhuB, fhuC*, and *fhuD* were the three proteins shared by these two complexes. In netC, module 37 consisted of four proteins, of which *ugpA, ugpB, ugpC*, and *ugpE* were exactly the four proteins that made up the glycerol-3-phosphate/glycerol-2-phosphate ABC transporter complex. As shown in Fig. 1c, module 76 and module 112 were perfectly matched with the *YhdW/YhdX/YhdY/YhdZ* ABC transporter complex and the galactofuranose /galactopyranose ABC transporter complex, respectively. The analysis above indicated that ELPA is an effective method to predict *E. coli* protein complexes in PPI network.

### Prediction of the functional module

**Fig. 2:**
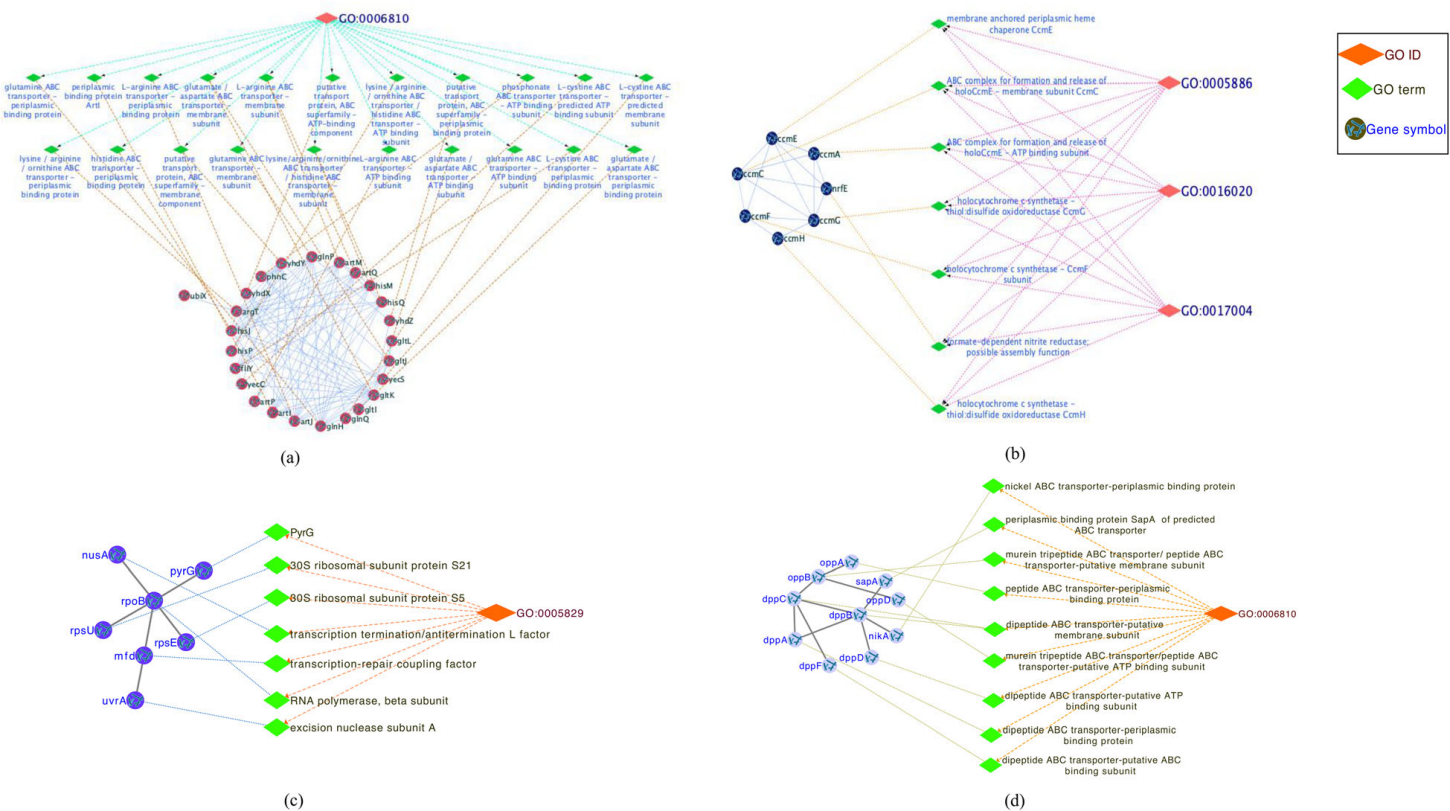
Predicted functional modules in netH and netC. **a** The proteins of module 19 in netH matched with GO annotations. **b** The proteins of module 40 in netH matched with GO annotations. **c** The proteins of module 9 in netC matched with GO annotations. **d** The proteins of module 26 in netC matched with GO annotations

Those protein modules of netH and netC identified by ELPA were matched with benchmark functional annotations of *E. coli* in the EcoCyc dataset, respectively. If the Matching Score between a protein module and a given GO term was greater than 0.5, this protein module was recognized as a potential functional module. In netH, most prediction modules (82.5%) were significant functional modules, of which about 30% exactly matched a certain functional term (Matching Score equal to 1). For example, as shown in Fig. 2a, module 19 contained 25 proteins, of which 24 proteins matched GO: 0006810. Obviously, it was a functional module. All the seven proteins of module 40 were completely covered by GO:0005886, GO:0016020, and GO:0017004, respectively (as shown in Fig. 2b). In netC, 91.6% of predicted modules were significant functional modules, of which 53.2% were completely covered by at least one GO term. For example, as shown in Fig. 2c and d, module 9 contained seven proteins: *mfd*, *nusA*, *pyrG*, *rpoB*, *rpsE*, *rpsU,* and *uvrA*, which were completly annotated by GO:0005829. Similarly, ten proteins of module 26: *dppA*, *dppB*, *dppC*, *dppD*, *dppF*, *nikA*, *oppA*, *oppB*, *oppD,* and *sapA* were all annotated with GO:0006810. The analysis above indicated that ELPA is an effective method to predict *E. coli* functional modules in PPI network.

### Comparative analysis of predicted protein complexes and functional modules

A protein complex is a physical aggregation of several proteins that interact at the same time and location through molecular interactions. A functional module also consists of multiple proteins that interact with each other to control or perform a particular cellular function. However, unlike protein complexes, these proteins do not necessarily interact at the same time and location. Therefore, the comparative analysis of protein complexes and corresponding functional modules is of great scientific significance.

**Fig. 3:**
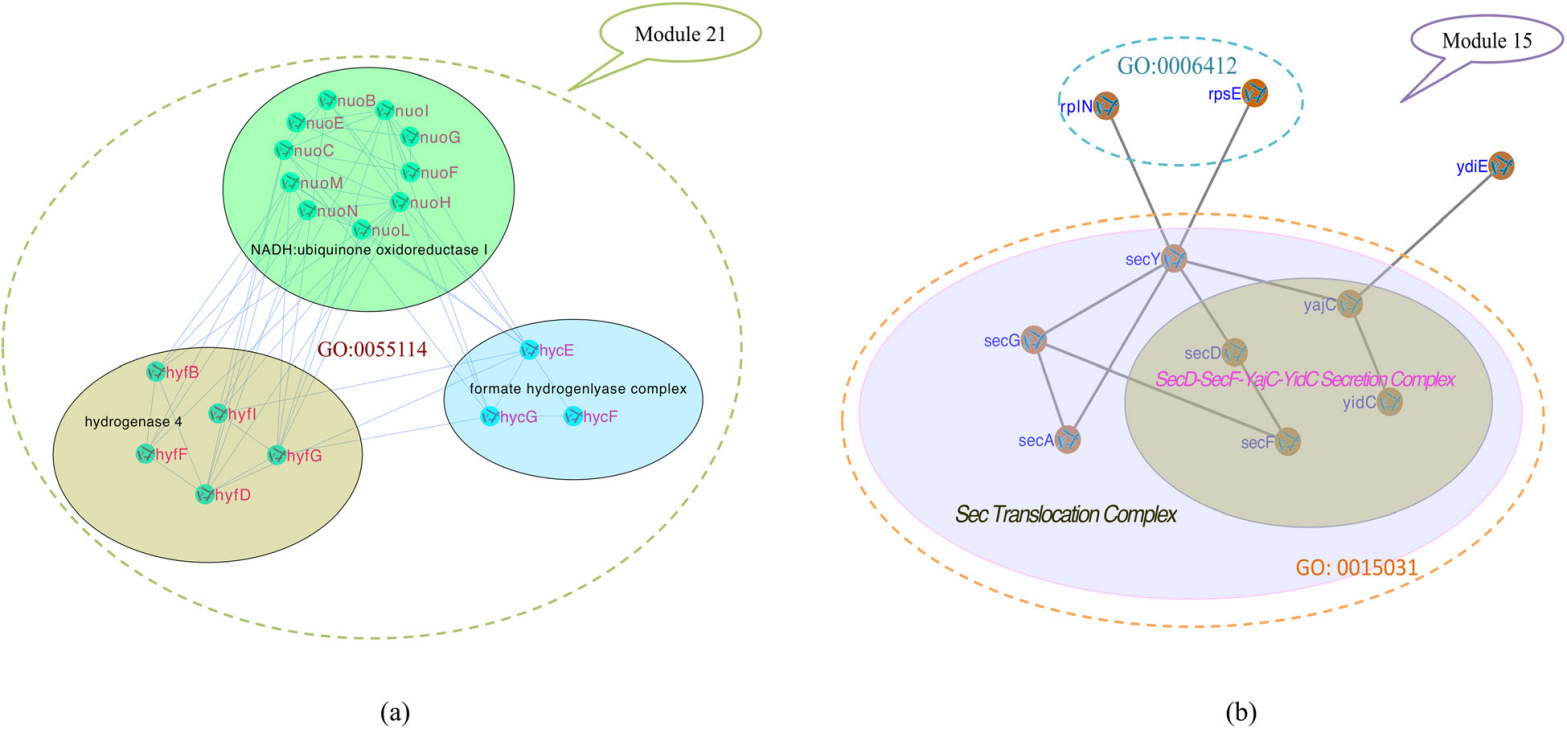
Correlation between protein complexes and functional modules. **a** Module 21 in netH covered by three real complexes, and fully annotated by GO:005514. Proteins functions in this module were all related to the above three real complexes. **b** Module 15 in netC mainly covered by maltose ABC transporter complex and fully annotated by three functions. Proteins functions in this complex were all related to the maltose ABC transporter

For example, as shown in Fig. 3a, module 21 in NetH was mainly composed of three real complexes: NADH: ubiquinone oxidoreductase I, hydrogenase 4 and formate hydrogenlyase. All proteins in the hydrogenase 4 complex were completely covered by this module, 10 out of 11 proteins of ubiquinone oxidoreductase I complex matched this module, and 3 out of 5 proteins of formate hydrogenlyase complex matched this module. Also, we noticed that GO: 0055114 completely covered the 18 proteins of this module, which meant that the three protein complexes mentioned above might combine to perform a certain function. *hycE*, *hycF* and *hycG* were the components of the formate hydrogenlyase complex, and their functional annotations were hydrogenase 3, formate hydrogenlyase complex iron-sulfur protein, and hydrogenase 3 and formate hydrogenlyase complex-HycG subunit, respectively. This meant that the functions of these three proteins were consistent with the formate hydrogenlyase complex, and it also implied that the hydrogenase 3 complex was related to the formate hydrogenlyase complex. The Hydrogenase 4 complex proteins: *hyfB, hyfD, hyfF, hyfG* and *hyfI* were annotated with hydrogenase 4-component B, D, F, and large, small subunit respectively. This indicated that the functions of these five proteins and the hydrogenase 4 complex were highly coherent. The ten remaining proteins of this module: *nuoB, nuoC, nuoE, nuoF, nuoG, nuoH, nuoI, nuoL, nuoM,* and *nuoN* were all related to NADH: ubiquinone oxidoreductase complex. Therefore, we predicted that the above three protein complexes might significant functional correlations.

Figure 3b showed another similar case. Module 15 in netC consisted of ten proteins and contained two real complexes: *Sec* Translocation Complex and *SecD*-*SecF*-*YajC*-*YidC* Secretion Complex. All four proteins of *SecD*-*SecF*-*YajC*-*YidC* Secretion Complex were covered by this module and *Sec* Translocation Complex, and they were all matched with GO:0016021, among them *yidC* were annotated with inner-membrane protein insertion factor. 7 out 9 proteins of *Sec* Translocation Complex matched this module, and the seven proteins: *secA*, *secD*, *secF*, *secG*, *secY*, *yajC* and *yidC* were enriched with the term “protein transport” of GO: 0015031, which meant their functions were consistent with the complex. Besides one uncharacterized protein *ydiE*, the two remaining proteins *rplN* and *rpsE* were enriched with the term “translation” of GO: 0006412, and the term “rRNA binding” of GO:0019843, respectively. The results above indicated that those predicted protein modules are significant related to corresponding protein complexes and functional modules. Usually, a functional module might cover more than one protein complex.

### Comparison with MCL

Most biological network module identification methods were based on node clustering, and among them, MCL has been proven to be superior to other methods in identifying the protein modules in most cases [23, 26, 27]. ELPA is a module identification method based on link clustering. It considered the attitudes of nodes and links at the same time, and can better reflect the network structure than nodes [21, 28]. ELPA is a parameter-free method, and MCL used the default parameters. And the clustering results of ELPA and MCL were compared in both networks, respectively.

**Fig. 4:**
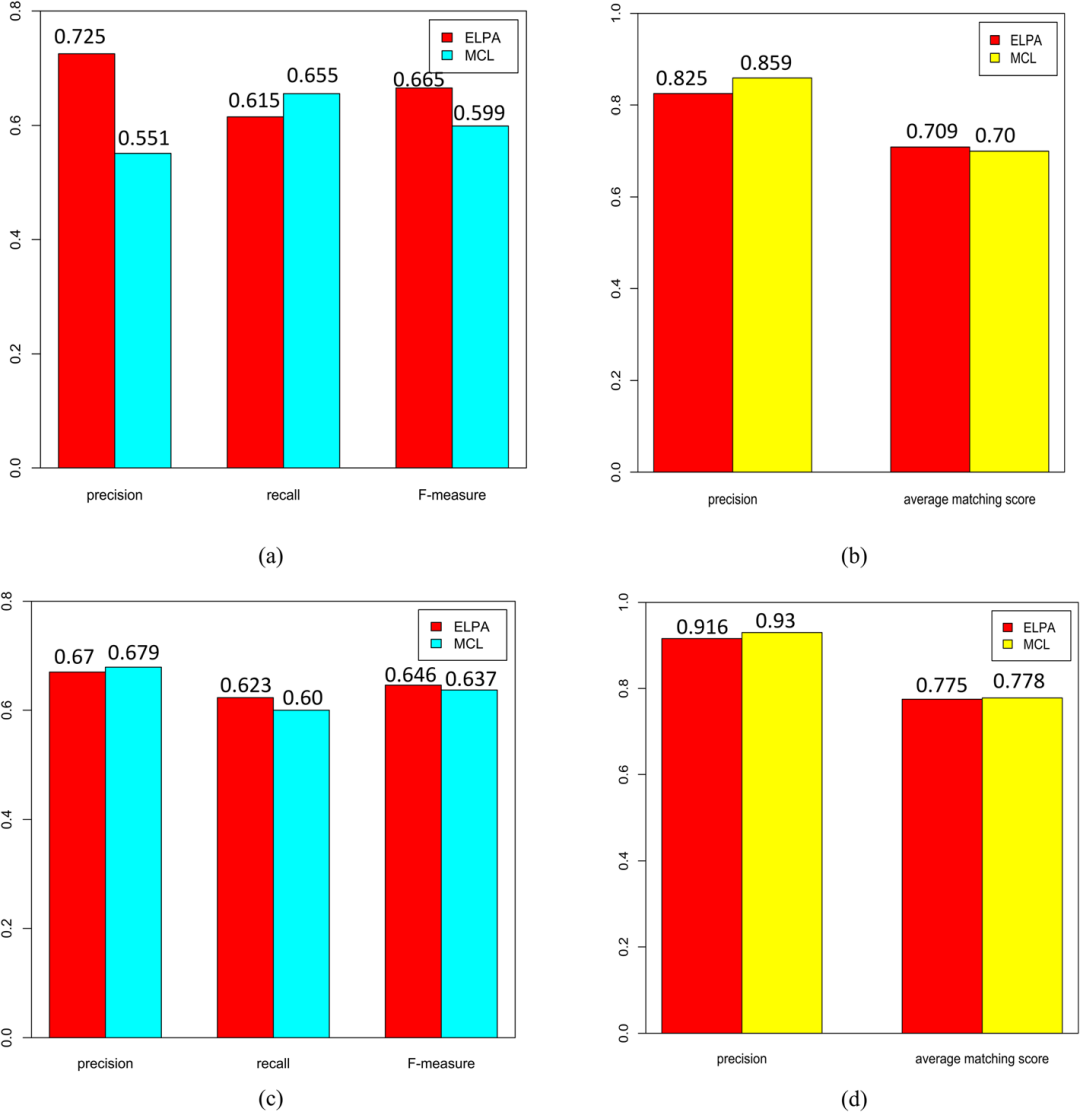
Comparative performance of ELPA and MCL in netH and netC. **a** Comparison of protein complex predictions in netH. **b** Comparison of functional modules predictions in netH. **c** Comparison of protein complex predictions in netC. **d** Comparison of functional modules predictions in netC

Three metrics: *Precision*, *Recall*, *F-measure* were utilized to compare the performance of MCL and ELPA in predicting protein complexes. In netH, the performance comparison of two methods for predicting protein complexes was shown in Fig. 4a. The values of *Precision*, *Recall* and *F-measure* of ELPA were 72.5, 61.5, and 66.5%, respectively, while the corresponding results of MCL were 55.1, 65.5, and 59.9%, respectively. Similar results were obtained in netC. As shown in Fig. 4c, the values of *Precision*, *Recall* and *F-measure* of ELPA were 67, 62.3 and 64.6%, while the corresponding results of MCL were 67.9, 60 and 63.7%, respectively. From the Fig. 4, we found that the performance of ELPA and MCL is comparable in the prediction of protein complexes. For example, in netH, Flagellum complex matched with module 10 of ELPA and module 59 of MCL, and the corresponding matching scores were 58.9 and 43%, respectively. Enterobactin synthase complex matched with module 53 of ELPA and module 46 of MCL, and corresponding matching scores were 50 and 45%, respectively. *SecD*-*SecF*-*YajC*-*YidC* Secretion complex matched with module 69 of ELPA and module 149 of MCL, and the corresponding matching scores were 56.3 and 45%, respectively.

In general, *Precision* and *AMS* are two effective metrics to evaluate the predicted quality of functional modules. The performance comparison of two methods for predicting functional modules was shown in Fig. 4b. Values of *Precision* and *AMS* of ELPA were 82.5 and 70.9%, respectively, and that of MCL were 85.9 and 70%, respectively. Analogous results were obtained in netC. As shown in Fig. 4d, values of *Precision* and *AMS* of ELPA were 91.6 and 77.5%, while the corresponding results of MCL were 93 and 77.8%, respectively. We concluded that the performance of both methods is comparable in the prediction of functional modules. For example, in netC, both module 190 of ELPA and module 66 of MCL were enriched in the GO: 0000018, and corresponding matching scores were 100 and 66.7%, respectively. Both module 29 of ELPA and module 101 of MCL were enriched in the GO: 0006281, and corresponding matching scores were 100 and 75%, respectively. Both module 83 of ELPA and module 168 of MCL were enriched in the GO: 0009060, and the corresponding matching scores were 80 and 75%, respectively. The results above showed that ELPA is an effective method to predict *E. coli* protein complexes and functional modules.

## Discussion

Besides well-characterized protein complexes and functional modules, we also identified modules that had not been matched to an EcoCyc protein complex or functional category in both networks. In netH and netC, we discovered 36 and 67 novel protein complexes, and 21and 17 novel functional modules, respectively (Table S2). A notable example of a novel protein complex was module 36 in netH, composed of five proteins: *pheS*, *pheM*, *thrS*, *argS* and *erfK*. *ThrS* is also a translational repressor protein, and it controls binds its own mRNA in the operator region upstream of the start codon. *ThrRS* represses translation by preventing the ribosome from to mRNA, and tRNA acts as an antirepressor allowing fine level control of enzyme synthesis. *ThrS*, *argS*, *pheS* and *pheM* are all involved in aminoacyl-tRNA ligase activity, and they are the members of arginine-tRNA ligase, phenylalanine-tRNA ligase alpha chain, phenylalanyl-tRNA synthetase operon leader peptide and threonine-tRNA ligase superfamily, respectively. In addition, *pheS*, *thrS* and *argS* are in Aminoacyl-tRNA biosynthesis pathway, and *pheM* is significant related to Aminoacyl-tRNA biosynthesis. We knew little about erfK except that it was involved in the peptidoglycan biosynthesis pathway. These annotations indicated that these proteins might constitute a protein complex involved in aminoacyl-tRNA biosynthesis.

An example for the novel functional module was module 10 in netC, and it consisted of seven proteins: *rpsN*, *rpmC*, *rplV*, *rlmJ*, *sbcB*, *holC* and hflC. *RpsN* binds 16S rRNA, required for the assembly of 30S particles and may also be responsible for determining the conformation of the 16S rRNA at the A site. *RplV* binds specifically to 23S rRNA. Its binding is stimulated by other ribosomal proteins, and makes multiple contacts with different domains of the 23S rRNA in the assembled 50S subunit and ribosome. *RpmC* binds 23S rRNA, and contacts trigger factor. *RlmJ* specifically methylates the adenine in position 2030 of 23S rRNA, and it required for the utilization of extracellular DNA as the sole source of carbon and energy. *HolC* is part of the beta sliding clamp loading complex, which hydrolyzes ATP to load the beta clamp onto primed DNA to form the DNA replication pre-initiation complex. *SbcB* degrades single-stranded DNA (ssDNA) in a highly possessive manner, and also functions as a DNA deoxyribophosphodiesterase that releases deoxyribose-phosphate moieties following the cleavage of DNA at an apurinic/apyrimidinic (AP) site by either an AP endonuclease or AP lyase. *HflC* controls the lysogenization frequency of phage lambda. Together, these annotations suggested that these proteins form part of a translation module.

## Conclusion

In this paper, a link clustering algorithm (ELPA) was used to identify protein complexes and functional modules in the *E. coli* PPI network. Through comparison with the EcoCyc database, we have discovered some novel and interesting complexes and functional modules. In addition, we compared and analyzed protein complexes and functional modules derived from the same predicted protein modules. It was found that protein complexes are highly overlapped with the corresponding functional modules, many of which contain more than one protein complex, which helps to understand the dynamic relationship between protein complexes and functional modules. Finally, the results of ELPA were compared with that of MCL, and we found that their performance is comparable in most cases. Therefore, we concluded that ELPA can be used as an effective cluster analysis tool for different types of biological networks. In further work, we will explore the key regulatory proteins and pathways in the transcriptional regulatory network of *E. coli*, based on the corresponding protein complexes and functional modules.

## Methods

### Source of PPI data

Two high-throughput experiments of protein-protein interactions (PPIs) datasets of *E. coli* were retrieved from the original paper of Hu et al. [5] and Cong et al. [8], respectively. Most of the data from these resources came from Yeast-Two- Hybrid (Y2H) and Tandem Affinity Purification (TAP). The dataset provided by Hu et al. included a large-scale TAP-derived network and a functional network. Our analysis merged the two networks into a single combined network which contained 7613 interactions among 2283 proteins. The dataset recently published by Cong et al. is a large-scale Y2H-derived network of *E. coli*, which contained 1618 interactions among1, 476 proteins. To predict protein complexes more efficiently, only those binary interactions associated with known complex proteins were considered. As a result, 3280 interactions among 1298 proteins were retrieved from the dataset of Hu et al., and 1299 interactions among 785 proteins were retrieved from the dataset of Cong et al.

### Benchmark for protein complexes and functional annotations

**Fig. 5:**
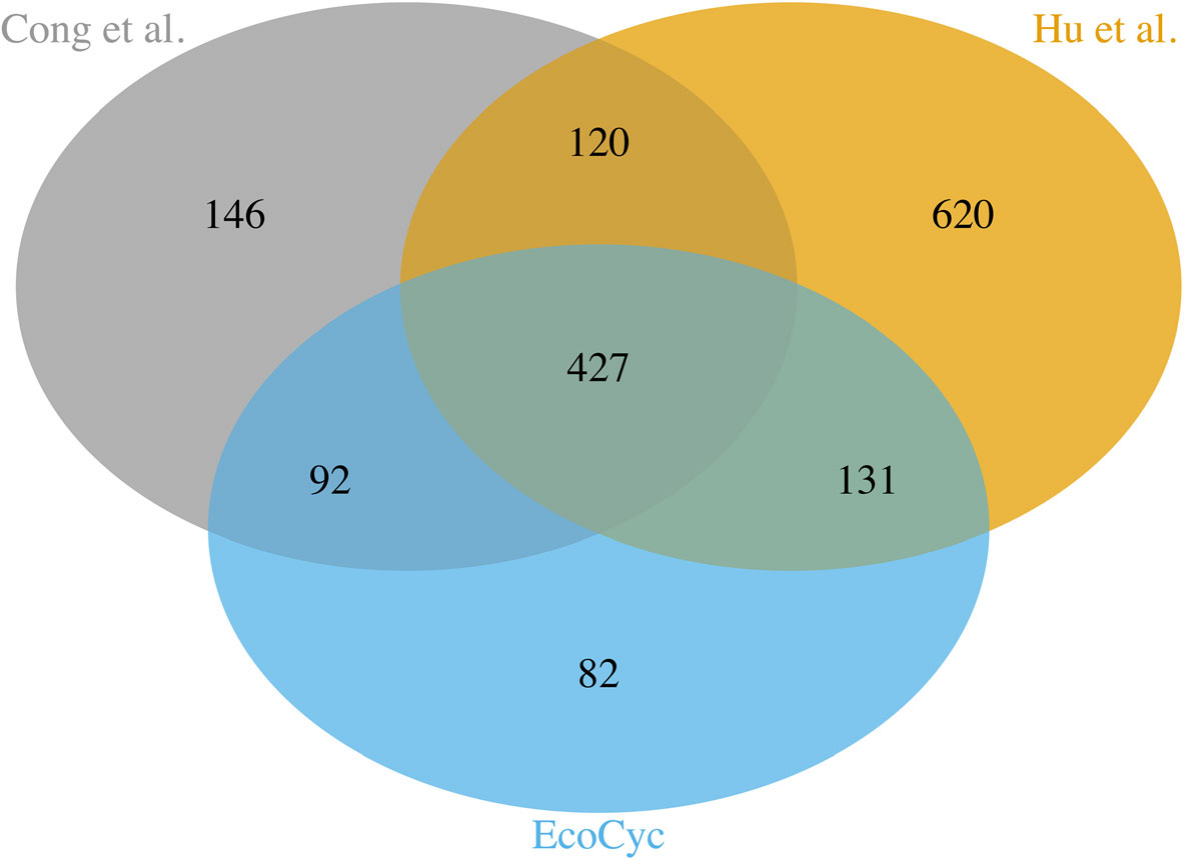
The relationships among three protein datasets (EcoCyc, Hu et al. and Cong et al.)

In most studies, protein complexes and functional annotations of *E. coli* downloaded from EcoCyc database [29] were regarded as the “gold standard”, and all these data sets downloaded are up-to-date. As we all know, many protein complexes of *E. coli* contain only two proteins, thus those complexes containing at least two proteins in the *E. coli* K-12 dataset of EcoCyc were kept as benchmark complexes. In this way, we obtained 295 benchmark protein complexes containing 732 proteins. Furthermore, the EcoCyc Gene Ontology (GO) database was taken as the benchmark functional classes of *E. coli*. In total, we obtained three protein datasets: EcoCyc, Hu et al., and Cong et al., and we found the relationships among them by Venn diagrams (shown in Fig. 5).

### Prediction of protein modules

In the past decade, many clustering algorithms for complex networks have been developed, most of which are based on node clustering. However, only a few methods can be used in complex biological networks. In this paper, an algorithm based on link clustering: Edge Label Propagation Algorithm (ELPA) [21], was utilized to identify the protein modules of *E. coli* in the above two PPI networks. The original paper [21] have shown that the performance of ELPA outperforms other link clustering algorithms [30, 31]. In contrast to node clustering, link clustering has the natural advantages of being compatible with the node attributes and link attributes of complex networks and can reflect the network topology structure better. Then, the protein modules detected by ELPA were matched with the “gold standard” protein complexes and functional annotations of EcoCyc and predicted the meaningful protein complex modules and protein functional modules.

### Evaluation methods

Two criteria were employed to evaluate the performance of ELPA. One was matching the identified protein modules with known protein complex of EcoCyc benchmark dataset. The other was the functional enrichment of the identified protein modules.

To determine the matching efficiency of a predicted complex *p* and corresponding real complex *b* in the benchmark complex set, the *Matching Score* (*MS*_*pb*_) between them was calculated as [32]:

1 $$ {MS}_{pb}=\frac{n_{pb}^2}{n_p\cdot {n}_b} $$

Where *n*_*pb*_ is the number of proteins shared by the predicted complex *p* and the real complex *b, n*_*p*_ is the number of proteins in complex *p,* and *n*_*b*_ is the number of proteins in complex *b*. A predicted complex and a real complex were considered to be a match if their matching score was no less than a specific threshold (typical threshold is 0.2 [24, 26]).

To evaluate the predicted protein complexes, we checked how well the predicted complexes matched the actual complexes. Three types of popular evaluation criteria: *Precision*, *Recall* and *F*-measure [28], were used to quantify the quality of the predicted protein complexes. Let *P* and *B* denote the set of predicted and actual complexes, respectively. Let *N*_*P*_ denote the number of protein complexes, and let *N*_*B*_ denote the number of actual complexes in the benchmark dataset. Let *N*_*PC*_ denote the number of predicted complexes that matched at least one real complex, and let *N*_*BC*_ be the number of actual complexes that matched at least one predicted complex. *Precision* and *Recall* were then defined as follows:

2 $$ Precision=\frac{N_{PC}}{N_P} $$

3 $$ Recall=\frac{N_{BC}}{N_B} $$

4 $$ F=2\times \frac{Precision\times Recall}{\left( Precision+ Recall\right)} $$

The arithmetic mean of matching score *(AMS)* was another metric to evaluate the predicted protein complexes.

To evaluate the functional enrichment of a predicted protein module, the matching score between a predicted protein module and a given GO term was used to estimate whether the proteins in the predicted module were enriched for the GO term. Then, *Precision* and *AMS* were used to evaluate predicted functional modules.

## Supplementary information

**Additional file 1.** S1-clustering results

**Additional file 2.** S2-novel complexes and functional modules

## Data Availability

All datasets used in this paper are public and public access to all databases is open. The data of Cong et al. is available at https://science.sciencemag.org/content/365/6449/185/tab-figures-data, the data of Hu et al. is available at http://compsysbio.org/bacteriome/download.php, and EcoCyc *E. coli* protein complexes and functional annotations dataset is available at https://ecocyc.org/.

## Abbreviations

*E. coli*: *Escherichia coli*
PPI: Protein-protein interaction
ELPA: Edge label propagation algorithm
MCL: Markov clustering algorithm
Y2H: *Yeast-Two- Hybrid*
TAP: *Tandem Affinity Purification*
*AMS*: *Arithmetic mean of Matching Score*
